# The effects of adding exogenous lignocellulose degrading bacteria during straw incorporation in cold regions on degradation characteristics and soil indigenous bacteria communities

**DOI:** 10.3389/fmicb.2023.1141545

**Published:** 2023-05-10

**Authors:** Yunlong Wang, Xuelian Zhang, Zixi Lou, Xiaoya An, Xue Li, Xinbo Jiang, Weidong Wang, Hongyan Zhao, Minjie Fu, Zongjun Cui

**Affiliations:** ^1^College of Agronomy, Yanbian University, Yanji, China; ^2^College of Agronomy and Biotechnology, China Agricultural University, Beijing, China; ^3^Heilongjiang Bayi Agricultural University, Daqing, China

**Keywords:** rice straw incorporation, composite microbial systems, microbial community structure, lignocellulose decomposition, cold regions

## Abstract

Low temperature is one of the bottleneck factors that limits the degradation of straw during rice straw incorporation. Determining strategies to promote the efficient degradation of straw in cold regions has become a highly active research area. This study was to investigate the effect of rice straw incorporation by adding exogenous lignocellulose decomposition microbial consortiums at different soil depths in cold regions. The results showed that the lignocellulose was degraded the most efficiently during straw incorporation, which was in deep soil with the full addition of a high-temperature bacterial system. The composite bacterial systems changed the indigenous soil microbial community structure and diminished the effect of straw incorporation on soil pH, it also significantly increased rice yield and effectively enhanced the functional abundance of soil microorganisms. The predominant bacteria SJA-15, *Gemmatimonadaceae*, and *Bradyrhizobium* promoted straw degradation. The concentration of bacterial system and the depth of soil had significantly positive correlations on lignocellulose degradation. These results provide new insights and a theoretical basis for the changes in the soil microbial community and the application of lignocellulose-degrading composite microbial systems with straw incorporation in cold regions.

## 1. Introduction

Straw incorporation can affect the soil organic matter and environment and crop yields (Wang et al., 2021). Straw degradation primarily relies on microbial decomposition in the soil. Soil microbes can improve the soil environment and increase crop yields by degrading lignocellulose and releasing nutrients into the soil (Passoth and Sandgren, 2019). Thus, it is important to explore the relationship between straw incorporation and soil microorganisms for the agriculture and environment.

It is well known that the soil microbial community is the core of straw degradation (Li et al., 2021). However, most studies on the interrelationship between straw incorporation and soil microorganisms have been limited to the effects of straw return on soil microorganisms. Previous studies introduced that straw affects the microbial community structure by changing the habitat of microorganisms (Wang et al., 2014), and straw incorporation significantly increased the diversity and relative abundance of soil microorganisms (Cong et al., 2020), it also increased significantly the total amount of soil microorganisms after straw incorporation (Liu X. et al., 2022). In contrast, few studies have been conducted to improve the effectiveness of straw incorporation by modifying the soil microbial community. In general, straw incorporation significantly changes the soil microbial community, but this does not solve the problem of inefficient degradation of straw by indigenous microorganisms. Therefore, we tried to explore this problem by reverse thinking, we improved the degradation efficiency of straw by changing the community structure of indigenous bacteria.

Straw incorporation is affected by several factors, overall, the root causes of the differences in straw degradation are owing to effects on the soil microbial community (Fierer et al., 2003). The soil microorganisms vary significantly at different soil depths (Song et al., 2022). Under normal circumstances, although deep soil has fewer resources, the soil environment is stable. In contrast, shallow soil has abundant resources but is more affected by the environment (Griffiths et al., 2003). This is one of the reasons for the differences in microbial communities at different soil depths (Wu et al., 2022). Moreover, there is a direct relationship between microbial abundance and the degradation of lignocellulose (Meng et al., 2022). Therefore, enhancing the degradation efficiency of straw by improving the structure of indigenous soil microbial communities are promising approaches.

Previous studies have shown that the addition of exogenous composite bacterial systems can change the indigenous microbial community structure to some extent, which means that increasing significantly the abundance and diversity of predominant bacteria and maintaining long-term stability (Chen et al., 2021; Nigussie et al., 2021). Recent studies have shown that exogenous composite microbial systems can be directly applied to incorporated straw. Phukongchai et al. (2022) reported that the incorporation of straw with the addition of microorganisms that can degrade lignocellulose accelerated the decomposition of straw in the soil and significantly increased the activity of indigenous soil microbes. Thus, the addition of exogenous complex bacterial systems during straw incorporation would be more effective at degrading straw and causing significant differences in microbial communities. To date, there has been little research about the incorporation of straw in different soil depths with the addition of high-and low-temperature composite microbial systems in cold regions.

Based on the findings described above, this study aimed to investigate the effect of composite microbial systems on straw degradation in different soil depths and their effect on the soil microbial community structure. For this purpose, we established two soil depths (35 cm and 15 cm) and two composite microbial systems (high and low temperature) to return straw to the field. The process was conducted under low temperatures in the winter, and the efficiency of straw incorporation was studied by measuring the lost of lignocellulose weight. The composition of the soil active microbial community was analyzed by 16S rRNA gene sequencing. In this manner, the effects of the composite bacterial systems on the effects of straw incorporation and the soil microbial community were revealed to provide a new theoretical basis for straw incorporation and the application of composite bacterial systems in cold regions.

## 2. Materials and methods

### 2.1. Sample source

Soil samples were taken from the experimental site of the College of Agriculture, Yanbian University, Yanji, China (latitude 129°49’N, longitude 42°92′E). The site is located in a high latitude basin in the middle temperate zone with a temperate monsoon climate, average annual precipitation of 583 mm, average annual temperature of 7.1°C, lowest temperature of –31.4°C, frost-free period of 146 d throughout the year, and soil type of chalky clay loam.

Straw samples were taken from the experimental base of the College of Agriculture, Yanbian University. After the crop was mechanically harvested, straw pieces that were approximately 10 cm long were screened and sampled, subsequently air-dried, and stored for backup.

The low-temperature composite consortium is a stable composite bacterial system designated PLC-8 that was sampled from the soil of perennial straw piles during low temperatures. After continuous passage culture, it is highly effective at decomposing lignocellulose at 20°C, and the predominant genera include *Chryseobacteria*, *Brevundimonas*, and *Acinetobacte*r (Cui et al., 2021). However, the degradation efficiency of low-temperature composite consortium is generally lower than that of high-temperature composite consortium. Thus so we also chose the high-temperature composite consortium for this study. The high-temperature composite consortium was MC1, which was selected and combined from the compost samples. It can be kept stable by continuous passage culture, and it was highly effective at decomposing lignocellulose at 50°C; the predominant genera were *Clostridium*, *Pseudoxanthomonas*, *Brevibacillus*, and *Bordetella* (Hua et al., 2014).

### 2.2. Experimental design

Ten grams of air-dried straw were weighed into a nylon mesh bag (specifications: 0.15 mm aperture, 15 cm long, and 10 cm wide), while 10 g of soil was added to the bag to increase the contact area between the straw and soil. The nylon bag was placed at the corresponding treatment soil depth, and the compound bacterial system was then quantitatively applied on the surface of the nylon bag and finally filled with soil.

In this experiment, there were 10 treatments, and each treatment was repeated three times. The soil depth, concentration, and type of complex strains in each treatment are shown in Table 1. The concentration of complex bacteria was established as described by Phukongchai et al. (2022), and the total test time was 270 d. Distilled water was added regularly to maintain the soil moisture, and no sampling was conducted of straw during the decomposition of straw until the end of sampling test. The soil was sampled on 0 d, 90 d, 180 d, 270 d, and determined the soil pH and soil organic matter without extensive damage to the soil environment around the straw, and the soil organic matter was determined by the potassium dichromate method. At the end of the straw incorporation, rice was transplanted into the treated soil and two rice were planted at each treatment site to determine the yield.

**Table 1:** Type and concentration of the composite microbial system at different depths of straw incorporation, the slash line indicates not added.

Group	Straw incorporation depth (cm)	Composite microbial system volume fraction (%)	Composite microbial system type
DH5	35	50	High temperature
DH2	35	25	High temperature
DL5	35	50	Low temperature
DL2	35	25	Low temperature
DCK	35	/	/
SCK	15	/	/
SH5	15	50	High temperature
SH2	15	25	High temperature
SL5	15	50	Low temperature
SL2	15	25	Low temperature

DH5 means Deep soil with High temperature consortiums and the volume fraction was 50%, DH2 means means Deep soil with High temperature consortiums and the volume fraction was 25%, DL5 means Deep soil with Low temperature consortiums and the volume fraction was 50%, DL2 means Deep soil with Low temperature consortiums and the volume fraction was 25%, DCK means control check group in Deep soil. SCK means control check group in Shallow soil. SH5 means Shallow soil with High temperature consortiums and the volume fraction was 50%, SH2 means Shallow soil with High temperature consortiums and the volume fraction was 25%, SL5 means Shallow soil with Low temperature consortiums and the volume fraction was 50%, SL2 means Shallow soil with Low temperature consortiums and the volume fraction was 25%.

### 2.3. Lignocellulose analysis

The straw was dried and sieved. The hole was 1 mm in diameter, and the straw was placed in special filter bags for neutralization and acid washing. The content of hemicellulose was determined by a lignocellulose analyzer (ANKOM 200i; ANKOM Technologies, Macedon, NY, United States). The samples were washed with 72% concentrated sulfuric acid and ashed in a muffle furnace (DHX-12-1,200; GUANGSHU Mechanical and electrical equipment co., LTD., Shang Hai, China) to determine the contents of cellulose and lignin. The amount of weight that was lost from each component of the lignocellulose was calculated.

### 2.4. Determination and analysis of the soil microbial sequences

After the experiment, The soil in the nylon mesh bag and the 2 mm soil sample on the surface of the nylon mesh bag were extracted and the soil samples were well mixed. The DNA from soil was extracted, and the genes were sequenced by Shanghai Meiji Biomedical Technology (Shanghai, China). The V3-V4 region of the bacterial 16SrRNA gene was amplified, and the primers were the universal bacterial primers 338F (5′-ACTCCTACGGGAGGCAGCAG-3′) and 806R (5′-GGACTACHVGGGTWTCTAAT-3′). The samples were sequenced on an Illumina MiSeq platform (San Diego, CA, United States).

### 2.5. Statistical analysis and model building

A bioinformatics analysis was performed using the Majorbio cloud platform. The abundance at each taxonomic level was analyzed by QIIME (version 1.9.1), and the β-diversity distance was calculated. Uparse v. 7.0 and USEARCH v. 7.0 were used to cluster the operational taxonomic units (OTUs) and perform statistical analysis. An α-diversity analysis was conducted using Mothur v. 1.30. The data were collated and preliminarily mapped using igraph, Hmisc, pheatmap, and vegan package in the R 4.2.1 environment. Structural equation models (SEMs) were created for the data, and the model was constructed using SPSS 20 and Amos 25 (IBM, Inc., Armonk, NY, United States).

## 3. Results

### 3.1. Effect of the addition of lignocellulose decomposition microbial consortiums on the rate of lignocellulose degradation during the process of straw incorporation

**Figure 1:**
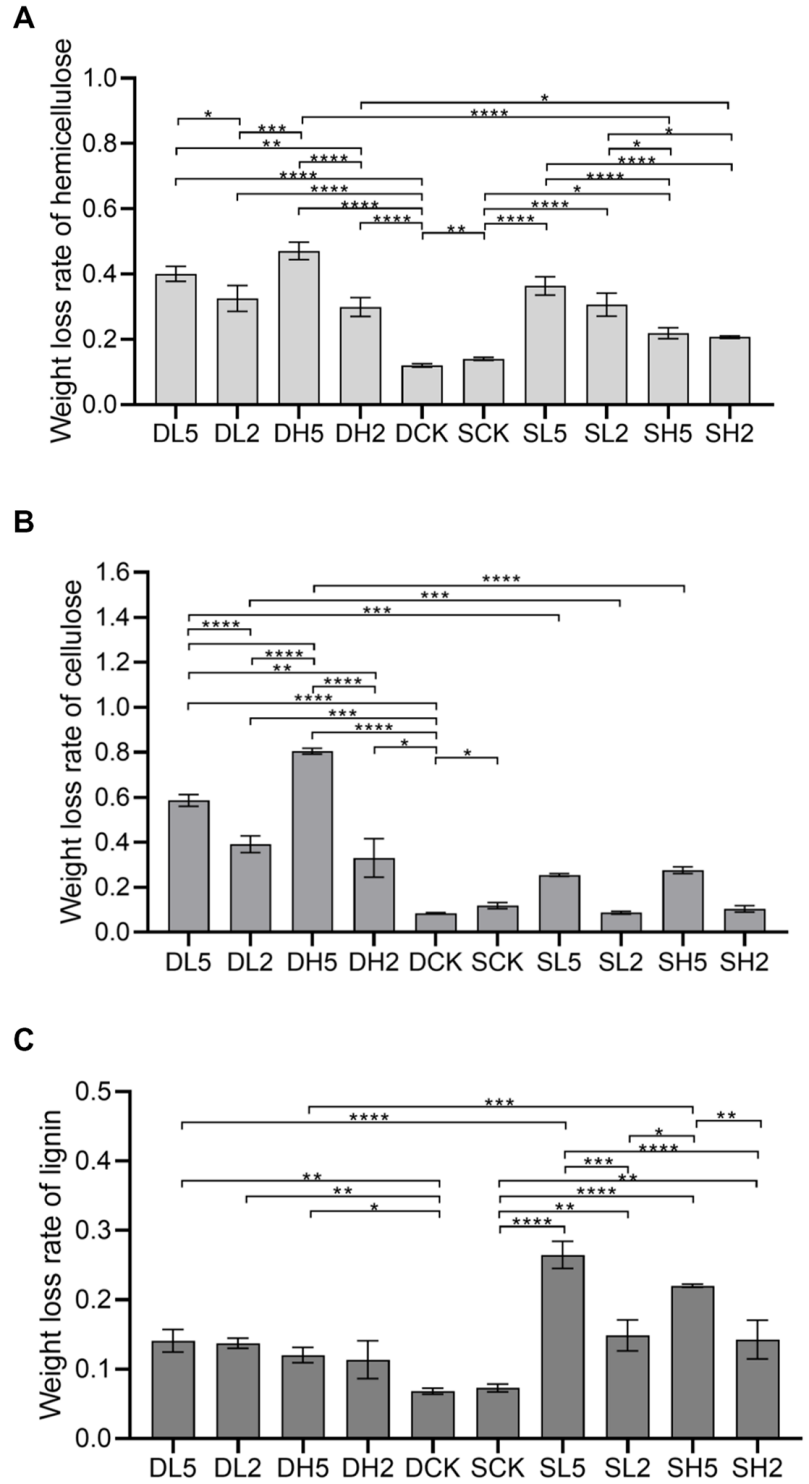
The rate of weight lost from hemicellulose, cellulose and lignin in different groups. **(A)** Weight lost rate of hemicellulose, **(B)** weight lost rate of cellulose, **(C)** weight lost rate of lignin. Significance levels: PERMANOVA, ^*^p < 0.05, ^**^p < 0.01, ^***^p < 0.001, ^****^p < 0.0001.

Lignocellulose is primarily composed of hemicellulose, cellulose, and lignin. The degree of degradation of these three components characterizes the straw degradation effect (Shinde et al., 2022). The highest amount of weight lost from hemicellulose was 47.11%, which was observed for DH5 as shown in Figure 1A. This rate was significantly higher by 35.05% compared with that from the DCK. At the same soil depth, the amount of weight lost from hemicellulose of DH5 was significantly higher by 17.19% compared with DH2 and 14.58% compared with DL2. However, it did not differ significantly from that of DL5. In different depths, the amount of weight lost from hemicellulose of DH5 was significantly higher than that of SH5 by 25.18%. The weight lost by SCK was higher than that of DCK (without exogenous microbiota) The highest amount of cellulose of 80.53% was lost by DH5 as shown in Figure 1B, which was significantly higher than that of DCK by 71.98%, DH2 by 47.49%, and DL5 by 21.97%. The weight of cellulose lost by DH5 was significantly higher than that of SH5 by 52.94% at different soil depths. Moreover, the amount of cellulose lost by SCK was higher than that of DCK (without exogenous microbiota). A relatively low amount of lignin was lost because its structure is more complex, which renders biodegradation difficult (Meng et al., 2022). The highest weight of lignin lost was a decrease of 25.09%, which was observed for SL5 as shown in Figure 1C. This amount was significantly higher by 17.74% compared with that of the SCK, 11.79% compared with SL2, and 12.79% compared with SH2. However, there was no significant difference between SL5 and SH5. The rate of lignin weight lost increased significantly by 10.99% in SL5 compared with DL5 at different depths. There was no significant difference between DCK and SCK. Overall, deep soils were more effective at degrading cellulose and hemicellulose with the addition of exogenous microorganisms than shallow soils, and shallow soils were more effective at degrading lignin, which could be related to the structure of the microbial community in soil and the functional species expressed.

### 3.2. Effect of the addition of lignocellulose decomposition microbial consortiums on the pH during the process of straw incorporation

**Figure 2:**
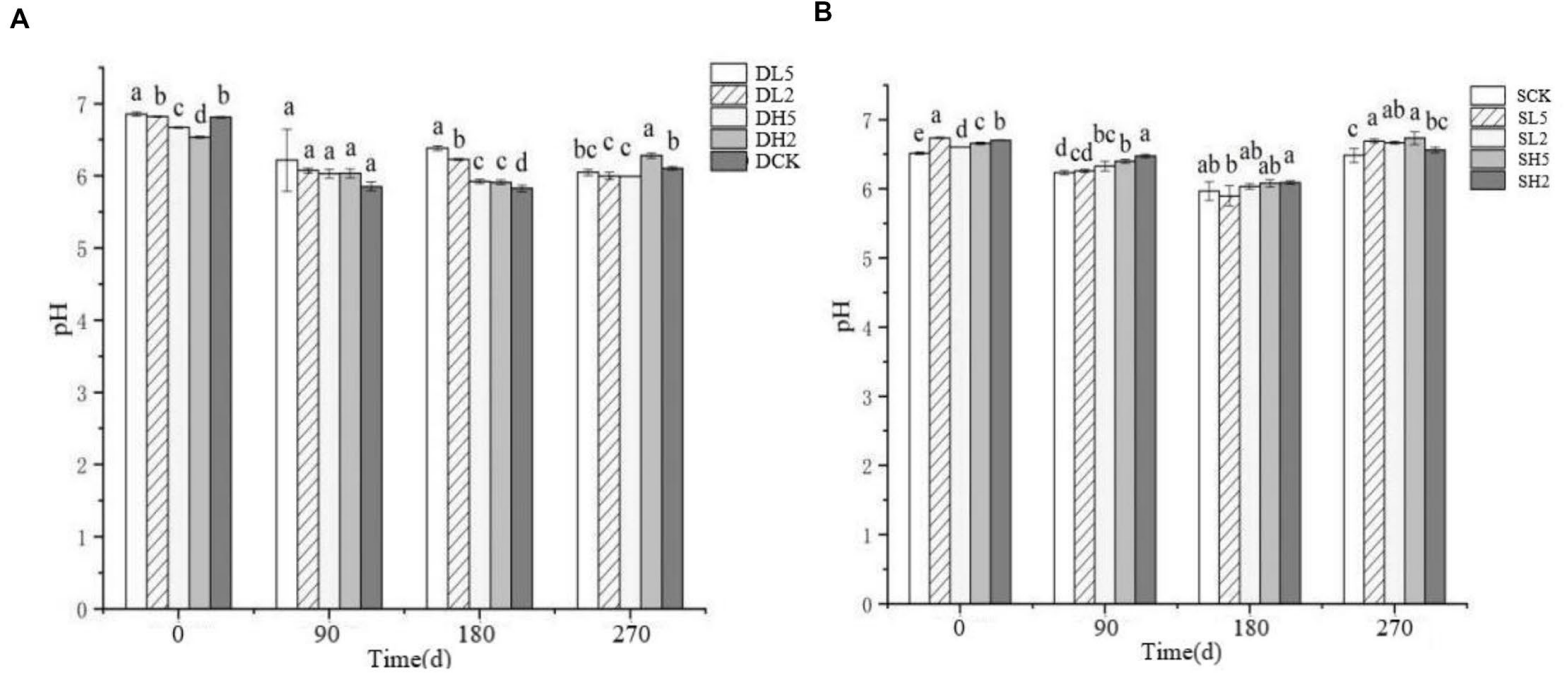
Change of pH in different treatment groups. **(A)** The treatments in deep soil. **(B)** The treatments in shallow soil, Significance: PERMANOVA, *p* < 0.05.

The effect of straw incorporation with exogenous microbial consortiums in deep soil on pH is shown in Figure 2A. The 0 d indicates that the soil pH was measured immediately after the straw was incorporated. The pH of DL5 on 0 d was greater than that of the other treatment groups. There was no significant difference between DL2 and DCK, and the pH of DH2 was less than that of the other treatment groups. The pH of all treatment groups decreased on 90 d. The pH of DL5 was greater than that of the other treatment groups on 180 d, and the pH of DCK was the smallest. On 270 d, the pH of DH2 was greater than the other groups, and the difference in pH between the other groups was small. The effect of shallow soil treatments on pH is shown in the Figure 2B. The pH of SL5 was greater than the other groups on 0 d, and there was little difference between the rest of the groups. The pH of SH2 was greater than that of other groups, and there was little difference between the other groups on 90 d. The pH of all groups decreased on 180 d, while they increased on 270 d. The pH of SH5 was the largest, and that of SCK was the smallest. Overall, straw incorporation decreased the soil pH to some extent. The treatment groups with the addition of exogenous microbial consortia had a higher pH value compared with the control group, which indicates that the addition of exogenous microbial consortia will decrease the effect of straw incorporation on the soil pH to some extent.

### 3.3. Effect of the addition of lignocellulose decomposition microbial consortiums on the soil organic matter during the process of straw incorporation

**Figure 3:**
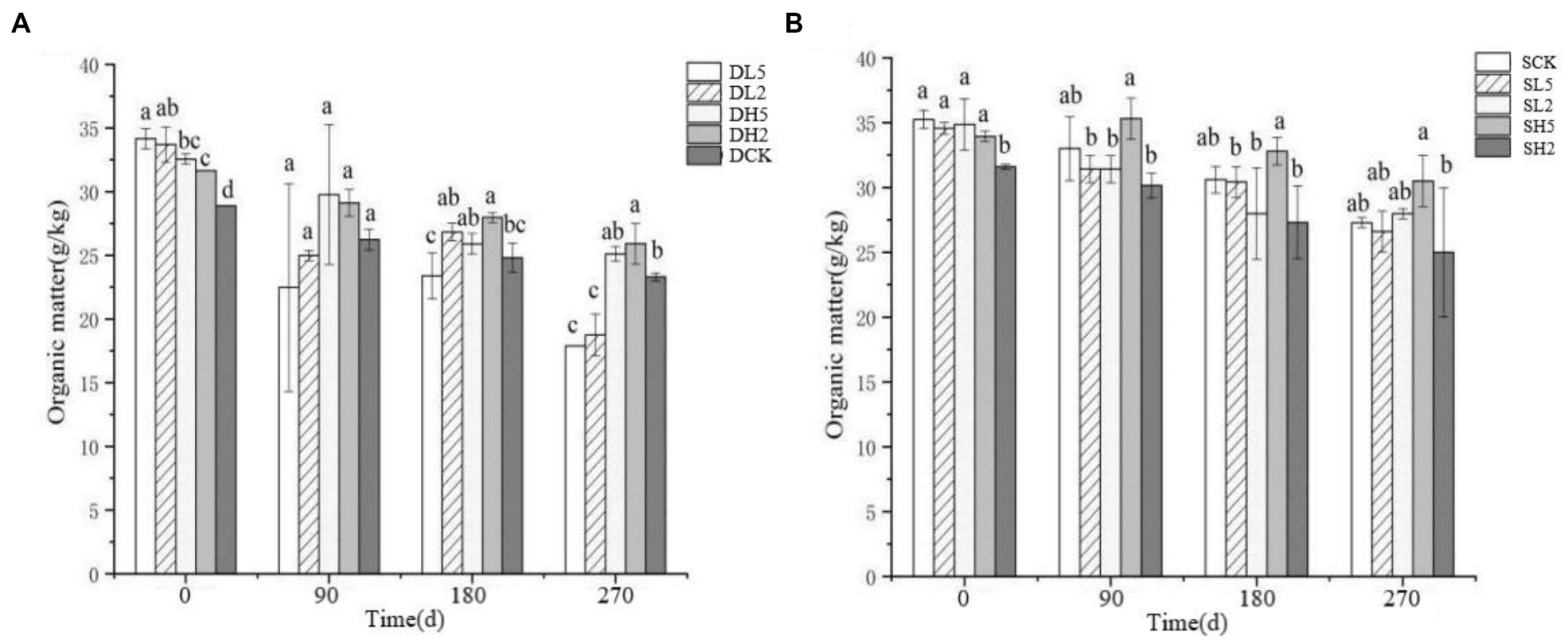
Change of soil organic matter in different treatment groups. **(A)** The treatments in deep soil. **(B)** The treatments in shallow soil, Significance: PERMANOVA, *p* < 0.05.

The effect of straw incorporation with exogenous microbial consortia on deep soil organic matter is shown in Figure 3A. The soil organic matter of DL5 was significantly greater than that of the other groups, and DCK had the least organic matter on 0 d. The soil organic matter of DL5 was smaller than that of the other treatment groups on 90 d. The soil organic matter of DH2 was larger than that of the other groups, and DL5 was the smallest on 180 d. The soil organic matter of DL5 and DL2 was significantly smaller than that of the other groups, and DH2 was the largest on 270 d. In the shallow treatments shown in Figure 3B, SH2 had the smallest soil organic matter, and there was no significant difference between the other groups on 0 d. SH5 had the largest content of soil organic matter compared with the other groups, and SH2 was the smallest on 180 d. SH2 had the smallest soil organic matter, and SH5 had the largest on 270 d. Overall, the soil organic matter content decreased as the treatment time increased. The addition of exogenous microbial consortia significantly reduced the content of soil organic matter in both deep and shallow soils, and deep soils were affected to a greater extent than shallow soils.

### 3.4. Effect of the addition of lignocellulose decomposition microbial consortiums on the yield during the process of straw incorporation

The effect of straw incorporation with exogenous microbial consortia on rice yield is shown in Supplementary Table S1. DH2 had the largest effective panicle number. SCK had the smallest effective panicle number. DH5 had the largest number of grains per panicle. SCK had the smallest number of grains per panicle. SH2 had the highest setting rate, and SCK had the lowest setting rate. DH5 had the largest 100-grain weight, while SCK had the smallest 100-grain weight. SH2 had the highest yield, and DCK had the lowest yield. Overall, the yields of all the treatment groups after the exogenous microorganisms were added were greater than those of the control group without the addition of microbial consortia, thus, indicating that the incorporation of straw with the addition of exogenous microbial consortia could significantly improve rice yields.

### 3.5. OTU clustering analysis of the microbial community during the process of straw incorporation

The flattening of the Shannon curves in Supplementary Figure S1A indicates the amount of genes sequenced was sufficient to reflect the majority of microbial information in the samples with a high degree of confidence. We found that the microbial abundance increased as the soil deepened which is consistent with the conclusions of a previous study (He et al., 2022). Meng et al. (2022) confirmed that low microbial abundance had a driving effect on the degradation of lignocellulose. Compared with the controls DCK and SCK, the abundance of each treatment group increased to varying degrees. Although DL2 had the highest abundance, it was not highly effective at decomposing lignocellulose. DH5 had 66 specific OTUs, and it was strongly effective at decomposing hemicellulose and cellulose. SL5 only had 58 specific OTUs, it had the highest amount of lignin lost (Supplementary Figure S1B). Moreover, combining the analysis of Supplementary Table S2 and Figure 1 indicated that the treatment groups with higher diversity indices did not have the strongest lignocellulolytic capacity.

### 3.6. PCA analysis of changes in the microbial community during the process of straw incorporation

**Figure 4:**
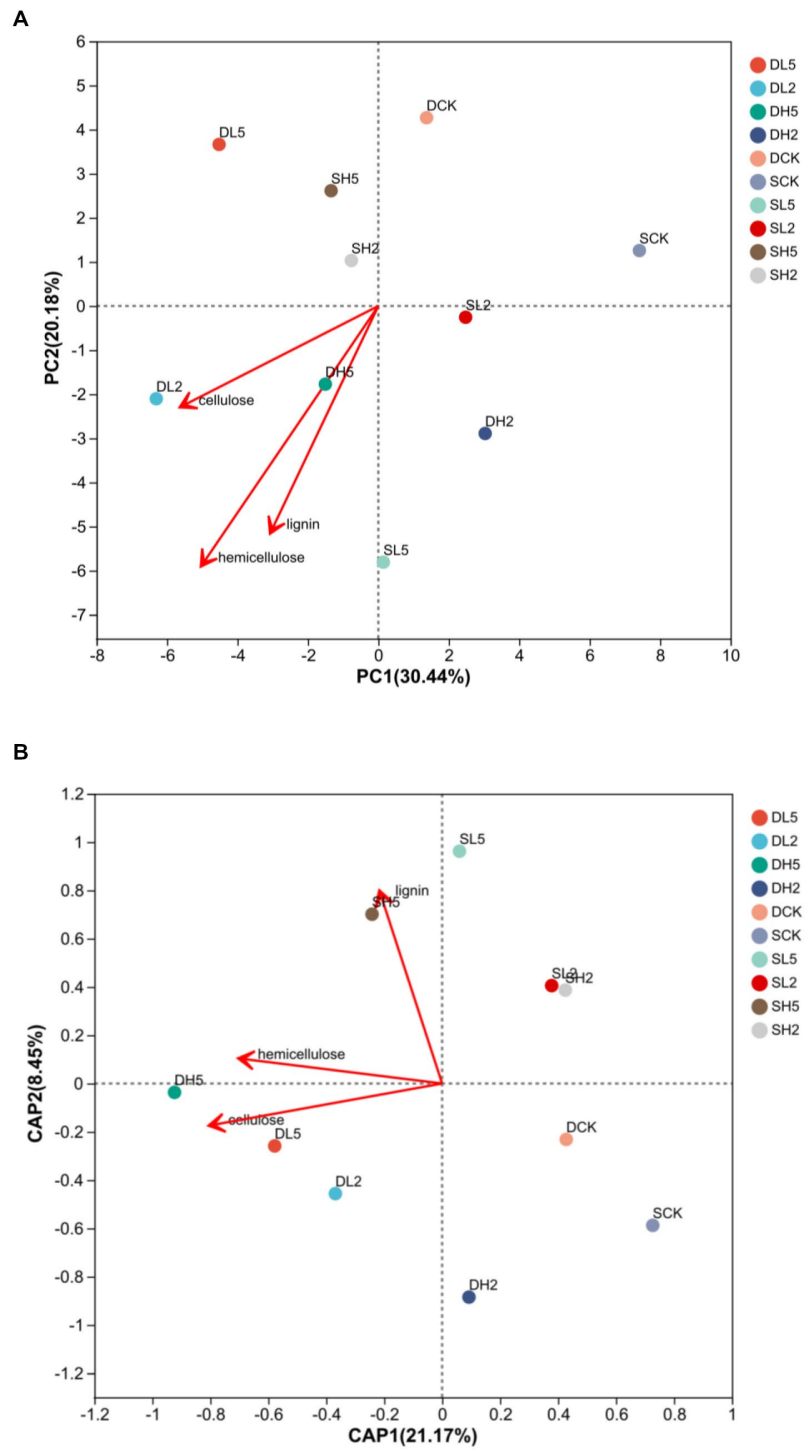
Principal coordinate analysis **(A)** and redundancy analysis **(B)** at the phylum level, different colors represent different treatments.

Figure 4A is a principal components analysis (PCA) was performed for all the treatments to determine the correlation of different groups with lignocellulose degradation. Compared with the other treatment groups, DH5 correlated the most highly with cellulose and hemicellulose, and SL5 correlated with lignin the most highly. Therefore, the microbial composition of DH5 was significantly correlated with the degradation of hemicellulose and cellulose. It was the most effective at degrading straw. Figure 4B is a distance-based redundancy analysis. After exogenous microorganisms were added, each treatment showed a positive correlation with lignocellulose decomposition, and also significantly higher than DCK and SCK. DH5 was significantly correlated with hemicellulose and cellulose decomposition, and SH5 was significantly correlated with lignin decomposition.

### 3.7. Analysis of the microbial community diversity in the straw incorporation

**Figure 5:**
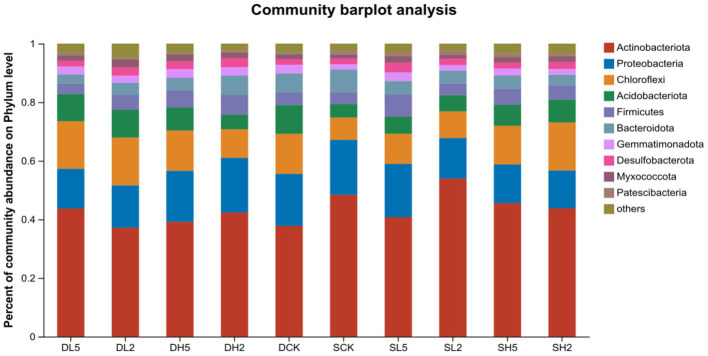
Community bar plot analysis on phylum level.

The stable microbial community composition in the soil after the straw incorporation is shown at the phylum taxonomic level in Figure 5. The dominant phyla were *Actinobacteriota*, *Proteobacteria*, *Chloroflexi*, *Acidobacteria*, *Firmicutes*, and *Bacteroidetes*, which accounted for 37.16 to 53.96%, 12.89 to 18.78%, 7.62 to 16.39%, 4.59 to 9.73%, 3.50 to 7.55%, and 3.15 to 7.84%, respectively. The abundance of *Firmicutes* was significantly higher in DH5 compared with that in DCK and *Chloroflexi* and *Acidobacteria* compared with DH2. This indicates that the abundance of microbial communities in DH5 differed significantly from those of both DH2 and DCK. The predominant bacteria in the high-temperature composite microbial system MC1 that was added were *Firmicutes* and *Proteobacteria*. These were more abundant in DH5. Previous studies suggest that *Firmicutes* plays a central role in lignocellulose degradation, and it primarily degrades hemicellulose (Gavande et al., 2021). *Proteobacteria* are associated with the degradation of lignocellulose, which entails the degradation of cellulose (Berlemont et al., 2014).

### 3.8. Analysis of the microbial community at the genus level following the addition of an exogenous composite bacterial system

**Figure 6:**
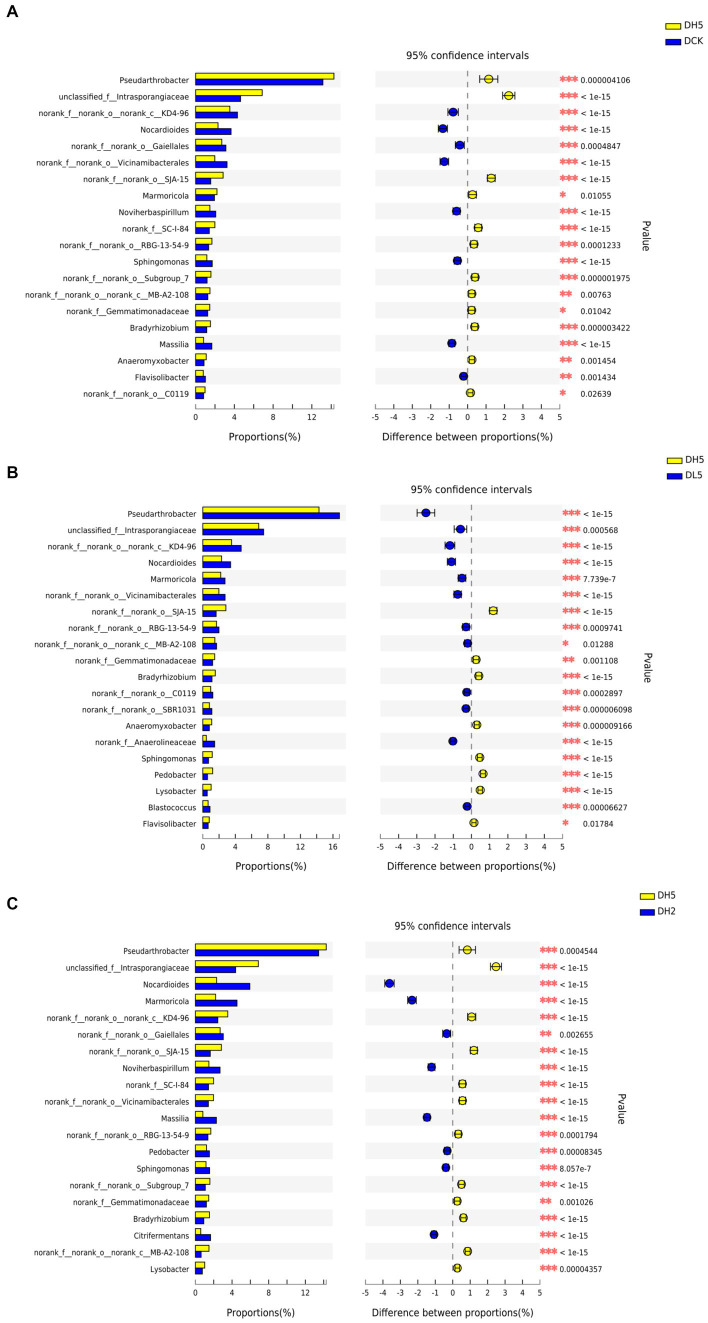
**(A)** DH5 and DCK, **(B)** DH5 and DL5, **(C)** DH5 and DH2 with the Fisher exact test bar plot on the genus level. ^*^p < 0.05. ^**^p < 0.01. ^***^p < 0.001.

There were significant differences in the genera contained in DH5 and those in the other treatments shown in Figure 6. SJA-15, *Gemmatimonadaceae*, and *Bradyrhizobium* were more prevalent in DH5 than in the other groups at the 0.05 level of significance. SJA-15 was reported to be an anaerobic bacterium capable of fermenting carbohydrates (Speirs et al., 2019). *Gemmatimonadaceae* can decompose cellulose (Frossard et al., 2021), and *Bradyrhizobium* primarily fixes nitrogen (Jin et al., 2022). The relative abundance of SC-I-84 in DH5 was greater than that of DCK and DH2, and related studies have shown that SC-I-84 is related to the content of elemental N in the soil (Yang et al., 2021). This further indicates that the decomposition of lignocellulose during the straw incorporation process consumes nitrogen sources in the soil. The relative abundance of *Pseudarthrobacter* and *Intrasporangiaceae* which of DH5 was greater than that of DCK and DH2. *Pseudarthrobacter* is associated with the degradation of hydrocarbons (Wang et al., 2019). *Intrasporangiaceae* can oxidize cellobiose and glucose under anaerobic conditions (Schellenberger et al., 2010). This analysis indicated that the microorganisms in DH5 were more effective at degrading lignocellulose than those in the other treatments.

### 3.9. Analysis of the relationship between the bacteria and lignocellulose degradation during straw incorporation

**Figure 7:**
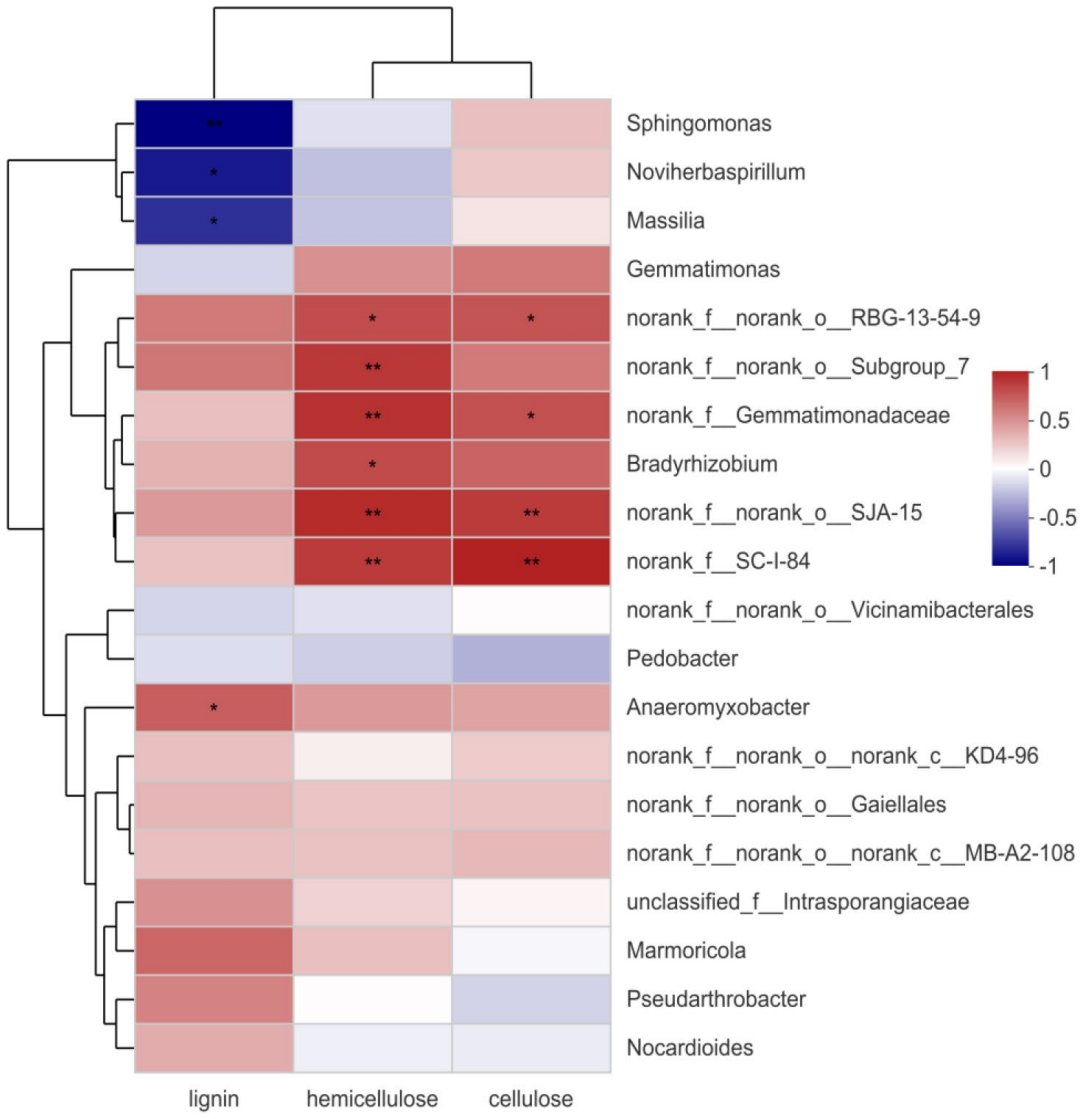
Spearman correlation heatmap. The red indicates positive correlation, blue indicates negative correlation. ^*^p < 0.05. ^**^p < 0.01.

Figure 7 shows the correlation between each bacterial genus and lignocellulose. *Gemmatimonadaceae*, RBG-13-54-9, SJA-15, and SC-I-84 significantly positively correlated with the decomposition of hemicellulose and cellulose. *Bradyrhizobium* only significantly positively correlated with hemicellulose, and *Anaeromyxobacter* significantly positively correlated with lignocellulose decomposition. *Anaeromyxobacter* has been reported to be a nitrogen-fixing bacterium (Masuda et al., 2020). Previous studies have confirmed the positive correlation between *Gemmatimonadaceae*, SJA-15, SC-I-84, *Bradyrhizobium,* and *Anaeromyxobacter* and the degradation of lignocellulose. This further indicates that the addition of a composite bacterial system plays an important role in the degradation of lignocellulose during straw incorporation, which is consistent with the analysis of its functional gene predictions.

### 3.10. FAPROTAX function gene prediction of the microbial community during straw incorporation

Analyzing the functions of microbial expression can help to determine the relationship between the efficiency of degrading lignocellulose and a composite bacterial system. Supplementary Figure S2 and Supplementary Table S3 indicated that DH5 had significantly higher abundances of hydrocarbon degradation and methylotrophy. The nitrogen fixation functions took place at a relatively high level. Although DH2 was the most effective at degrading xylan and cellulose, it was not the most efficient at degrading lignocellulose. This indicates that if the microbial system can degrade lignocellulose, additional hydrocarbon degradation, methylotrophy and nitrogen fixation will effectively increase the efficiency of lignocellulose degradation.

### 3.11. Relationship between the different factors and lignocellulose degradation by structural equation modeling

**Figure 8:**
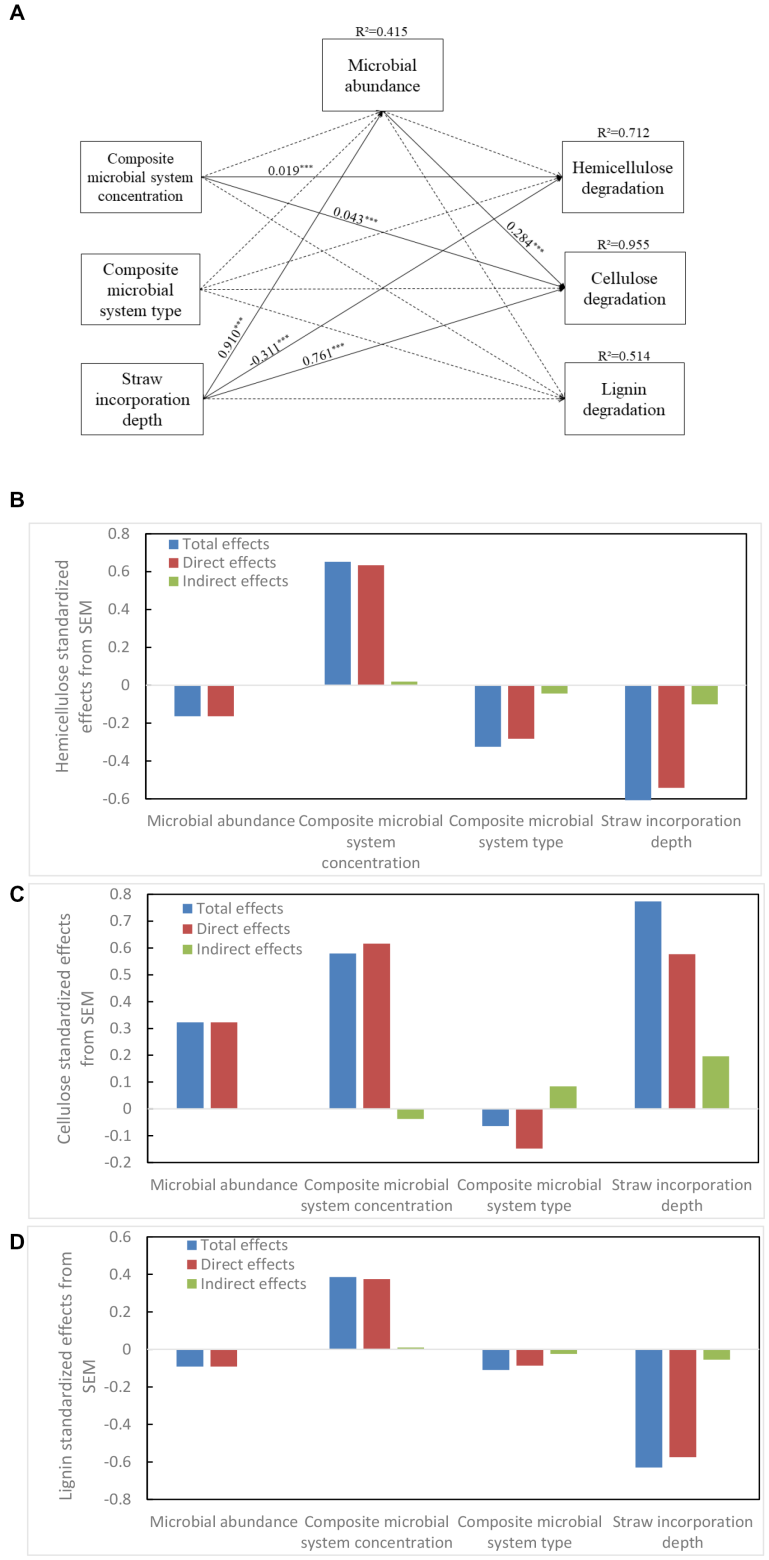
Structural equation models **(A)** show the direct and indirect effects of strain volume, strain type, soil depth, and microbial abundance on hemicellulose **(B)**, cellulose **(C)**, and lignin **(D)** degradation.

Structural equation modeling (SEM) is often used to quantify and compare the effective relationships between factors. In Figure 8, the concentration of composite bacterial system had a direct positive effect on the decomposition of hemicellulose and cellulose (λ = 0.019 ^***^, *p* < 0.05; λ = 0.043^***^, *p* < 0.001, respectively). There was no significant effect of composite bacterial type on each factor. The soil depth had a direct negative effect on the degradation of hemicellulose, and it had a direct positive effect on cellulose degradation; it also had a positive effect on soil microbial abundance (λ = −0.311 ^***^, *p* < 0.05; λ = 0.761 ^***^, *p* < 0.001, λ = 0.910 ^***^, *p* < 0.05, respectively). Soil depth had a positive effect on the degradation of cellulose (λ = 0.284 ^***^, *p* < 0.001, *R*^2^ = 0.955). Thus, soil depth had the greatest effect on lignocellulose followed by the concentration of composite bacterial systems. The depth of straw incorporation also had a strong effect on microbial abundance, which directly affected the efficiency of cellulose decomposition.

## 4. Discussion

Previous studies confirmed that the addition of composite microbial systems is an effective method to improve the structure of indigenous microbial communities (Win et al., 2021). We noted that DH5 and SL5 could effectively degrade lignocellulose, which significantly correlated with the functions of nitrogen fixation and hydrocarbon degradation. Our results indicated that not only is the degradation of straw directly influenced by the degradative functions of lignocellulose, but the degradation of lignocellulose is also promoted by nitrogen fixation, methylotrophy, and fermentation. While DH5 and SL5 had relatively high abundances of xylanolytic, cellulolytic, and ligninolytic functions, the abundances of nitrogen fixation, fermentation, methylotrophy, and hydrocarbon degradation functions were also significantly greater than those of DH2 and DL2, which led to a significant increase in the lost of lignocellulose weight in DH5 and SL5. A comparison of the differential strains in the samples showed that the abundance of SJA-15, *Gemmatimonadaceae*, and *Bradyrhizobium* was significantly higher in DH5 than in the other groups, and it was reported that SJA-15 can degrade lignocellulose (Shakeri Yekta et al., 2022). *Gemmatimonadaceae* is considered to be a denitrifying bacteria (Xiao et al., 2022). In the SL5, *Marmoricola*, *Nocardioides*, *Gaiellales*, *Anaeromyxobacter*, *Gemmatimonadaceae*, and *Bradyrhizobium* were significantly more abundant than those in the other groups, and *Marmoricola*, *Nocardioides*, and *Gaiellales* have been reported to be able to degrade lignocellulose (Schumann et al., 2018; Shi et al., 2020). *Anaeromyxobacter, Gemmatimonadaceae*, and *Bradyrhizobium* primarily fix nitrogen (Masuda et al., 2020). These results demonstrate that auxiliary strains significantly contribute to the degradation of lignocellulose, and nitrogen-fixing strains play an important role in the degradation of lignocellulose.

Our results demonstrated significant differences in the rates of weight lost of hemicellulose, cellulose, and lignin in different soil depths. Hemicellulose and cellulose decomposed more effectively in deeper soils after composite microbial systems were added, but the decomposition of lignin reacted in the opposite manner. More lignin was generally degraded in shallow soil layers compared with deeper ones after composite microbial systems were added. Previous studies have shown that deeper soil had lower levels of oxidation of lignin (Dao et al., 2018). Especially when after straw incorporation, the decomposition of lignocellulose was more efficient in surface soils than in deep soil (Zhang et al., 2019). It means the initiation of lignin turnover is more sensitive in surface soils than in deeper soils (Ma et al., 2022). These results demonstrate that the shallow soil will be more effective at degrading lignocellulose than the deep soil without adding exogenous composite microbial systems. However, the lignocellulose weight lost of all the treatments was greater than that of the control group after the exogenous composite microbial systems had been added, and DH5 lost the most lignocellulose weight in the deep soil. Thus, this demonstrated that the incorporation of straw in deep soil layers would be more efficient at degrading lignocellulose than the shallow soil layer after the exogenous composite microbial system had been added back.

An analysis of the community composition showed that bacteria at the genus and phylum level have been reported to be facultatively anaerobic, and a previous study also delineate this issue (Wang et al., 2018). The phylum taxonomic level, including *Actinobacteriota*, *Proteobacteria*, *Chloroflexi*, *Firmicutes*, *Gemmatimonadota*, and *Myxococcota* (Jiang et al., 2020; Murphy et al., 2021; Liu R. et al., 2022; Mujakić et al., 2022; Pan et al., 2022; Subirats et al., 2022), and the family and genus level, including *Pseudarthrobacter*, *Intrasporangiaceae*, and *Marmoricola* (Karaevskaya et al., 2021; Yu et al., 2022), all included facultative anaerobic strains. Therefore, facultative anaerobic bacteria may be an important influencing factor during the process of straw incorporation, and further research is merited to explore this issue.

## 5. Conclusion

The addition of complex bacteria systems significantly increased the amount of lignocellulose lost. The high temperature consortium effectively improved the efficiency of lignocellulose degradation. The composite bacterial systems significantly changed the microbial community structure in each treatment. It diminished the effect of straw incorporation on soil pH, and it also significantly increased rice yield and effectively enhanced the functional abundance of soil microorganisms. Therefore, when incorporating straw in cold areas, the addition of a complex microbial system is an effective treatment method.

## Data availability statement

The datasets presented in this study can be found in online repositories. The names of the repository/repositories and accession number(s) can be found in the article/Supplementary material.

## Author contributions

YW, HZ and MF designed the experiment. YW, XZ, ZL, XA, and XJ performed the experiment. YW and XL analyzed the data. YW wrote the manuscript. WW, HZ, MF and ZC revised the manuscript. XZ, HZ and MF evaluated the final version of the manuscript. All authors contributed to the article and approved the submitted version.

## Funding

This research was supported by the Special Fund for Agro-scientific Research in the Public Interest (no. 201503137) and the Department of Natural Science Fund Project of Jilin Province (no. YDZJ202201ZYTS578) and the Department of Key R&D Project of Jilin Province Science and Technology (no. 20200402040 N C).

## Conflict of interest

The authors declare that the research was conducted in the absence of any commercial or financial relationships that could be construed as a potential conflict of interest.

## Publisher’s note

All claims expressed in this article are solely those of the authors and do not necessarily represent those of their affiliated organizations, or those of the publisher, the editors and the reviewers. Any product that may be evaluated in this article, or claim that may be made by its manufacturer, is not guaranteed or endorsed by the publisher.
