# Supplementary material for: The effects of adding exogenous lignocellulose degrading bacteria during straw incorporation in cold regions on degradation characteristics and soil indigenous bacteria communities
Source: Front Microbiol. 2023 May 10;14:1141545. doi: 10.3389/fmicb.2023.1141545 (PMC10206022; doi:10.3389/fmicb.2023.1141545)
Supplement: Supplementary file 1 [file Data_Sheet_1.docx]

Supplementary Material

The effects of adding exogenous lignocellulose degrading bacteria during straw incorporation in cold regions on degradation characteristics and soil indigenous bacteria communities

Yunlong Wang^1#^, Xuelian Zhang^1#^, Zixi Lou^1^, Xiaoya An^1^, Xue Li^2^, Xinbo Jiang^1^, Weidong Wang^3^, Hongyan Zhao^1*^, Minjie Fu^1*^, Zongjun Cui^2^

***Correspondence:** Hongyan Zhao, [zhy@ybu.edu.cn](mailto:zhy@ybu.edu.cn)

Minjie Fu, fuminjie@163.com

**Supplementary Table S1. Yield of rice in different treatments. Significance: PERMANOVA, p < 0.05**

| Treatment | Panicle number | Number of grains per panicle | Setting rate (%) | Hundred-grain weight (g) | Yield (kg/ha) |
| --- | --- | --- | --- | --- | --- |
| DL5 | 31.3abc | 94.3a | 92.0a | 2.3b | 4402.5cd |
| DL2 | 31.0abc | 89.2a | 92.0a | 2.3b | 4584cd |
| DH5 | 34.0ab | 111.5a | 92.0a | 2.5a | 4738.5c |
| DH2 | 37.0a | 99.8a | 91.0b | 2.4a | 5451b |
| DCK | 21.6cd | 91.7a | 91.0b | 2.2a | 3010.5f |
| SCK | 17.6d | 81.6b | 86.0b | 2.0a | 3526.5e |
| SL5 | 20.0d | 89.2a | 91.0b | 2.3b | 4200d |
| SL2 | 19.0d | 93.4a | 92.0a | 2.3b | 4167d |
| SH5 | 20.3d | 104.9a | 92.0a | 2.2b | 5560.5b |
| SH2 | 21.8bcd | 97.6a | 95.0a | 2.2b | 6709.5a |


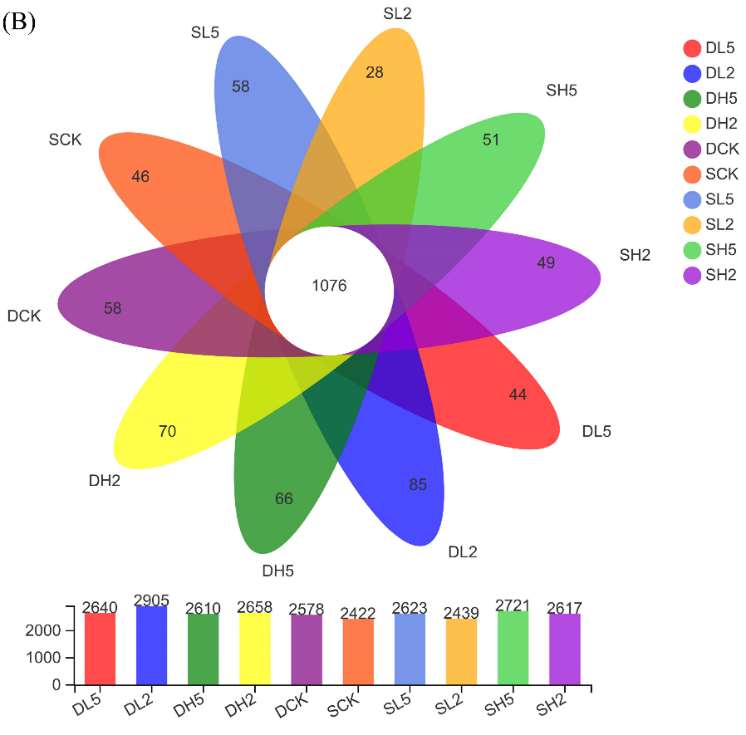

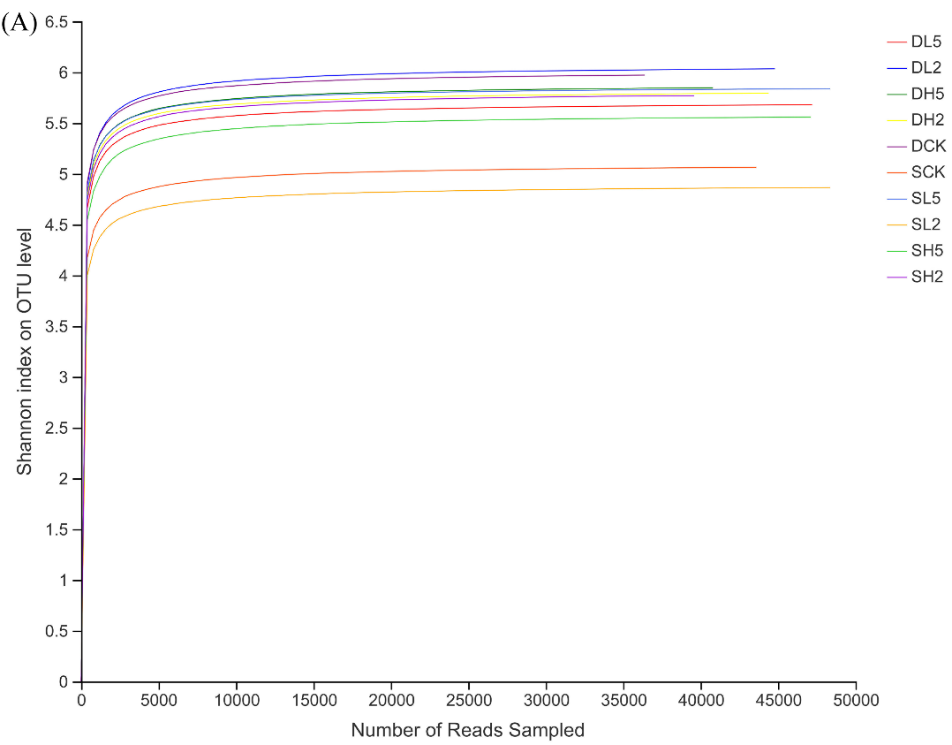


**Supplementary Figure S1.** **(A) Shannon curves in 16S rRNA sequencing, (B) Microbial Venn analysis**

**Supplementary Table S2.** **Diversity Index at OTU level. Significance: PERMANOVA, p < 0.05**

| Sample\Estimators | sobs | shannon | simpson | ace | pd |
| --- | --- | --- | --- | --- | --- |
| DL5 | 2640b | 5.679852b | 0.03347d | 3433.504ab | 212.0331ab |
| DL2 | 2905b | 6.033642a | 0.024241e | 3753.919ab | 231.764a |
| DH5 | 2610bc | 5.846972ab | 0.025304e | 3395.097ab | 206.2944ab |
| DH2 | 2658a | 5.794344b | 0.023169e | 3424.487a | 213.1432ab |
| abDCK | 2578bc | 5.971709a | 0.020992e | 3354.349ab | 208.5269ab |
| SCK | 2422bc | 5.062431c | 0.079285b | 3185.929ab | 202.2517b |
| SL5 | 2623c | 5.837524ab | 0.023656e | 3395.601ab | 209.4335ab |
| SL2 | 2439d | 4.864421c | 0.091602a | 3318.986b | 206.8161ab |
| SH5 | 2721bc | 5.557551bc | 0.046228c | 3588.226ab | 216.077ab |
| SH2 | 2617d | 5.769253b | 0.028211e | 3554.993ab | 212.6628ab |


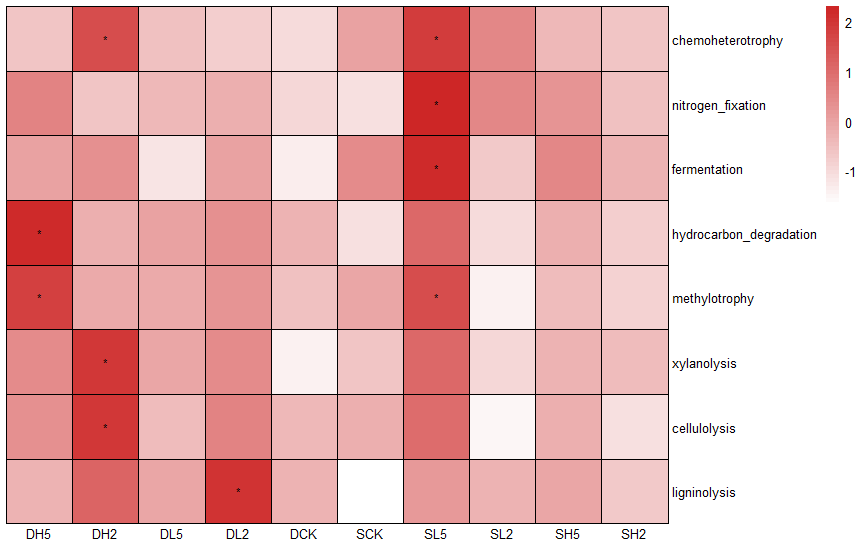


**Supplementary Figure S2.** **FAPROTAX functional predictions of different groups**

**Supplementary Table S3. FAPROTAX Function prediction index**

| Functional groups | DL5 | DL2 | DH5 | DH2 | DCK | SCK | SL5 | SL2 | SH5 | SH2 |
| --- | --- | --- | --- | --- | --- | --- | --- | --- | --- | --- |
| chemoheterotrophy | 7339 | 7004 | 7234 | 10577 | 6593 | 8210 | 11163 | 8951 | 7603 | 7272 |
| nitrogen_fixation | 868 | 902 | 1177 | 772 | 684 | 632 | 1731 | 1155 | 1079 | 808 |
| fermentation | 444 | 640 | 640 | 694 | 417 | 712 | 1015 | 524 | 722 | 595 |
| hydrocarbon_degradation | 255 | 278 | 436 | 232 | 226 | 160 | 338 | 163 | 234 | 187 |
| methylotrophy | 234 | 259 | 361 | 235 | 206 | 239 | 345 | 149 | 210 | 187 |
| xylanolysis | 10 | 13 | 13 | 23 | 1 | 6 | 17 | 4 | 8 | 7 |
| cellulolysis | 19 | 32 | 29 | 50 | 20 | 22 | 38 | 5 | 22 | 10 |
| ligninolysis | 11 | 18 | 12 | 22 | 11 | 4 | 13 | 11 | 12 | 9 |
